# Clinical Outcomes of Antrectomy for Patients With Multiple Type I Gastric Neuroendocrine Tumors: A Case Series

**DOI:** 10.1002/deo2.70363

**Published:** 2026-06-15

**Authors:** Yuki Morita, Ken Namikawa, Kaoru Nakano, Wataru Kurihara, Hiroyuki Yamamoto, Yusuke Horiuchi, Akiyoshi Ishiyama, Toshiyuki Yoshio, Souya Nunobe, Toshiaki Hirasawa

**Affiliations:** ^1^ Department of Gastroenterology Cancer Institute Hospital Japanese Foundation for Cancer Research Tokyo Japan; ^2^ Department of Gastroenterology Tonan Hospital Sapporo Hokkaido Japan; ^3^ Department of Internal Medicine Division of Gastroenterology Landspítali University Hospital Reykjavík Iceland; ^4^ Department of Pathology Cancer Institute Hospital Japanese Foundation for Cancer Research Tokyo Japan; ^5^ Division of Pathology Cancer Institute, Japanese Foundation for Cancer Research Tokyo Japan; ^6^ Department of Gastric Surgery Cancer Institute Hospital Japanese Foundation for Cancer Research Tokyo Japan

**Keywords:** antrectomy, autoimmune gastritis, endoscopic resection, gastric neuroendocrine tumors, regression

## Abstract

**Objectives:**

Type I gastric neuroendocrine tumors (T1‐GNETs) are frequently associated with autoimmune gastritis and typically present as multiple lesions. The treatment options for T1‐GNETs include endoscopic resection (ER), surgical resection, and pharmaceutical therapies. Antrectomy is reportedly an effective treatment for multiple T1‐GNETs; however, its efficacy and indication remain unclear. In this study, we aimed to assess the efficacy of antrectomy in patients with multiple T1‐GNETs.

**Methods:**

We retrospectively evaluated the clinical outcomes of eight patients with persistent multiple T1‐GNETs in the gastric remnant following antrectomy from 2005 to 2023.

**Results:**

All eight patients (median age: 60 years, male‐to‐female ratio 1:3) had body‐dominant atrophic gastritis. Median preoperative serum gastrin levels were 4800 (range, 2400–11,000) pg/mL. Preoperative endoscopy revealed <10 in two, 10–20 in one, and >20 in five patients. Antrectomy was performed as a primary surgery in three patients, as a completion surgery following non‐curative ER in two patients, and a combination therapy with pre‐antrectomy ER in three patients. Median serum gastrin levels decreased to 68 (range, 25–120) pg/mL during the median followup of 56 (range, 14–72 months). Tumor regression was observed in two patients and disappearance in six, with no recurrence or deaths.

**Conclusion:**

We assessed the residual T1‑GNETs post‐antrectomy and discovered either regression or disappearance in all cases. Normalized serum gastrin levels following antrectomy may have contributed to this outcome. On the basis of our findings, the combination of antrectomy and ER resulted in tumor regression that may represent a potential organ‐preserving approach for patients with multiple T1‐GNETs.

## Graphical abstract

Type I gastric neuroendocrine tumors (T1‑GNETs) often present as multiple lesions, complicating treatment. This retrospective case series study evaluates antrectomy outcomes in eight patients with persistent multiple T1‑GNETs during 2005–2023. Following antrectomy, tumor regression was observed in two patients and complete disappearance in six, with median serum gastrin levels decreasing from 4,800 to 68 pg/ml, during a median 56‐month follow‐up. No recurrences or deaths occur. Antrectomy combined with endoscopic resection represents a potential organ‐preserving approach for multiple T1‑GNETs.

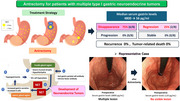

## 1 Introduction

Gastric neuroendocrine tumors (GNETs) are classified into three types according to the WHO classification [1]. Type I gastric neuroendocrine tumors (T1‑GNETs), accounting for 70%–80% of gastric NETs, are associated with chronic gastritis, primarily autoimmune gastritis [2, 3].

Management strategies for T1‑GNETs include endoscopic resection (ER), surgical resection, and pharmacological treatments [4, 5, 6, 7]. Total gastrectomy (TG) has historically been a commonly performed treatment since T1‑GNETs often present synchronous lesions [2, 6, 7]. However, it is highly invasive and may reduce postoperative quality of life (QOL). On the other hand, antrectomy, a treatment option in the Japanese and previous National Comprehensive Cancer Network (NCCN) guidelines, reportedly induces tumor regression or disappearance in gastric remnant by normalizing serum gastrin levels by removing gastrin‐secreting G‐cells in the pyloric glands [3, 7, 8, 9, 10, 11, 12]. Consequently, antrectomy allows patients with T1‑GNETs to avoid undergoing TG; however, the indications for its use are uncertain due to the limited number of reports [9, 13, 14, 15, 16, 17, 18]. ER is increasingly recognized as an effective option for T1‑GNETs, with growing evidence of indolent nature of T1‑GNETs and their favorable prognosis after local treatment [19, 20, 21, 22], but it has limitations when multiple lesions are present. Given the abovementioned advantages of antrectomy and ER, the combination of these two modalities may function synergistically in managing multiple T1‑GNETs. This study primarily aimed to evaluate efficacy of antrectomy in patients with multiple T1‑GNETs. Secondarily, we assessed the therapeutic potential of this combined approach.

## 2 Methods

### 2.1 Study Design

In this retrospective single‐center case series, we assessed the clinical outcomes of patients with residual T1‑GNETs following antrectomy for multiple T1‑GNETs treated at the Cancer Institute Hospital, Japanese Foundation for Cancer Research from 2005 to 2023. In this study, antrectomy was defined as distal gastrectomy (DG) including the antrum, regardless of lymph node dissection. The aim of antrectomy was to suppress gastrin production by resecting the antrum, which contains gastrin‐producing G cells [5, 23].

### 2.2 Diagnosis of T1‑GNETs

The diagnosis of T1‑GNETs was confirmed if the following criteria were met: (1) histological confirmation of neuroendocrine tumor; (2) presence of anti‐parietal cell antibodies (10‐fold increase or higher); (3) endoscopic findings meet the requirements for autoimmune gastritis (severe mucosal atrophy predominantly from the gastric body to the fundus is observed); (4) elevated serum gastrin (≥450 pg/mL); and (5) no evidence of gastrinoma [21, 22, 24].

### 2.3 Treatment and Surveillance Strategy

**FIGURE 1:**
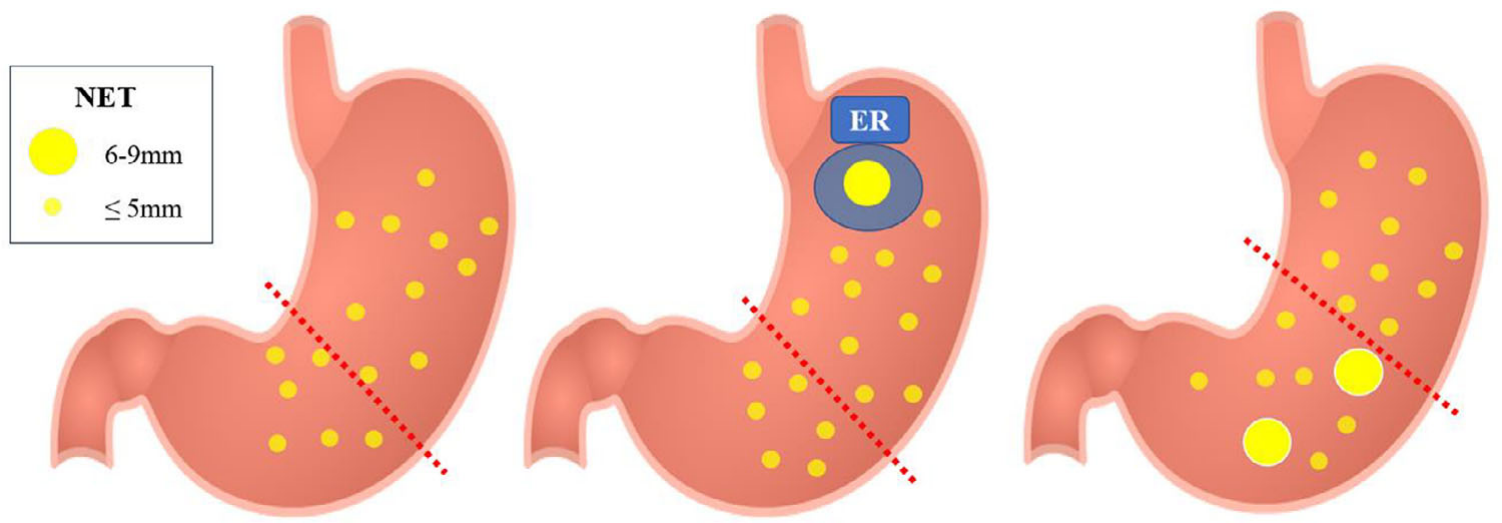
Treatment and surveillance strategy for selecting antrectomy. Post‐antrectomy, the residual tumor should be less than 5 mm. ER, endoscopic resection; NET, neuroendocrine tumors.

The treatment and surveillance strategy for patients with multiple T1‑GNETs in our institution included the following: (1) Tumors with diameters of <10 mm and an endoscopically manageable number were treated by ER. (a) If metachronous recurrence or tumor progression occurs during post‐resection surveillance, ER would be considered. (b) In cases with muscularis propria (MP) invasion or lymphovascular invasion (LVI), gastrectomy with lymph node dissection was performed as a completion surgery following non‐curative ER. (2) Tumors with diameters of ≥10 mm were resected by gastrectomy with lymph node dissection. (3) We considered antrectomy if ER for all tumors was challenging; however, there was no suspicious finding of MP invasion or lymph node metastasis. (a) Tumors with diameters of 6–9 mm located on the proximal side of the estimated resection area by antrectomy were removed using pre‐antrectomy ER (combination therapy with pre‐antrectomy ER). (b) Postoperative surveillance was conducted using endoscopy and CT scans. (c) The residual tumors of <5 mm were followed up post‐antrectomy, and if tumor progressed, ER would be considered. These strategies for selecting antrectomy are illustrated in Figure 1.

### 2.4 Assessment

We analyzed clinical, pathological, laboratory, and endoscopic data. They include age, sex, the presence and type of atrophic gastritis (corpus dominant, antral dominant, pangastritis, or none), serum gastrin levels, number of tumors, largest tumor size, depth of tumor invasion, LVI, lymph node metastasis, histological grading, follow‐up period after antrectomy, and postoperative clinical course. The number of lesions before treatment was determined on the basis of a combination of histological diagnosis from biopsy specimens and endoscopic evaluation, whereas the number of pathologically confirmed lesions was evaluated separately using the endoscopically and surgically resected specimens. Following antrectomy, endoscopic surveillance was performed first, and the assessment of tumor regression or disappearance relied primarily on endoscopic findings. Biopsy was additionally obtained only for lesions suspicious for residual NETs. The postoperative clinical course included the status of residual tumors, the period until disappearance of residual tumors, and rates of recurrence and mortality.

We evaluated the depth of tumor invasion using the Japanese Gastric Cancer Treatment Guidelines 2018 (5th edition) [25]. LVI and grading were evaluated using immunohistochemistry. Histological grading was evaluated according to the WHO classification [1].

## 3 Results

### 3.1 Clinical Characteristics of Patients

**FIGURE 2:**
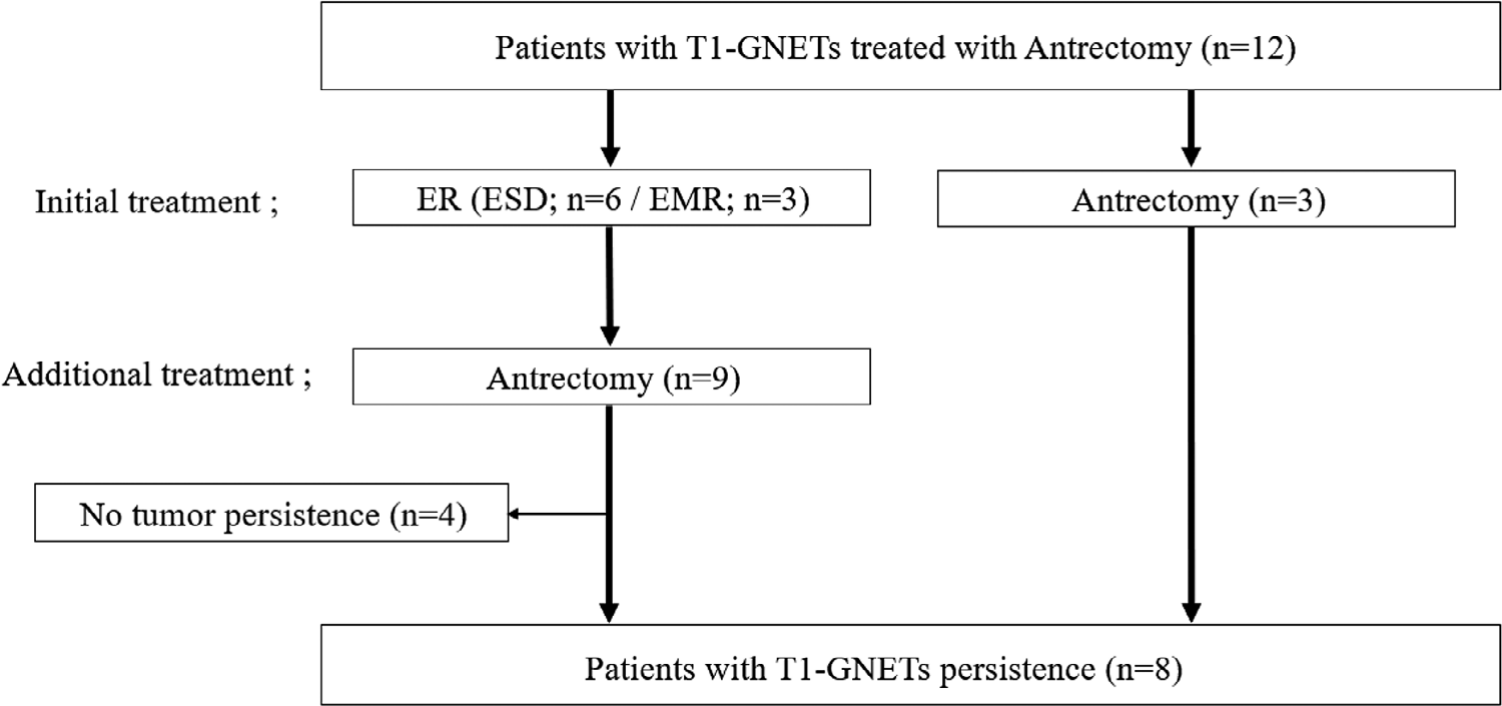
Patient flow diagram. T1‑GNETs, Type I gastric neuroendocrine tumors. EMR, endoscopic mucosal resection; ESD, endoscopic submucosal dissection; T1‑GNETs, type I gastric neuroendocrine tumors.

We found 12 patients with multiple T1‑GNETs who underwent antrectomy, with nine cases where antrectomy was performed as an additional treatment following ER. We excluded four cases without T1‐GNET in the residual stomach after antrectomy. We retrospectively analyzed the outcome of eight cases with residual T1‑GNETs following antrectomy during surveillance (Figure 2).

Table 1 summarizes the clinical characteristics of the eight patients. The median age of the patients was 60 years (range, 44–70 years), and the male‐to‐female ratio was 1:3. The region of atrophic gastritis was body‐dominant in all cases. The median serum gastrin levels of all patients were 4800 pg/mL (range, 2400–11,000 pg/mL). The number of tumors observed during endoscopy was <10 in two patients, 10–20 in one, and >20 in five. The median size of the largest tumor was 6 mm (range, 4–8 mm). Furthermore, antrectomy was performed as the primary surgery in three patients, as a completion surgery following non‐curative ER due to vascular invasion in two patients, and as a combination therapy with pre‐antrectomy ER in three patients.

**TABLE 1:** Clinical characteristics of patients.

	*n* = 8
Age, median (range), years	60 (44–70)
Sex, *n* (%)	
Male	2 (25.0)
Female	6 (75.0)
Atrophic gastritis, *n* (%)	
Corpus dominant	8 (100)
Antral dominant	0 (0.0)
Pangastritis	0 (0.0)
None	0 (0.0)
Serum gastrin levels, median (range), pg/mL Pre antrectomy	4800 (2400–11,000)
Number of tumors (whole stomach), *n* (%) �	
<10	2 (25.0)
10—20	1 (12.5)
>20	5 (62.5)
Size of largest tumor, median (range), mm �	6 (4–8)
Endoscopic resection, *n* (%)	
Yes	5 (62.5)
No	3 (37.5)
Treatment strategy, *n* (%)	
Antrectomy alone	3 (37.5)
Endoscopic resection and antrectomy as completion surgery following non‐curative ER	2 (25.0)
Combining pre‐antrectomy endoscopic resection and antrectomy	3 (37.5)

*Note*: �, endoscopic evaluation.

### 3.2 Pathological Characteristics of NETs in the Resected Stomach

Table 2 shows the pathological characteristics of NETs in the resected stomach. The number of tumors pathologically evaluated in the resected specimens was <10 in four patients, 10–20 in two, and >20 in two. In the most extensive case, 43 lesions were pathologically confirmed as NETs. The median diameter of the largest tumor was 5 mm (range, 1.5–8 mm). The depth of tumor invasion extended to the mucosa and submucosa in two and six patients, respectively. We observed LVI (Ly0, V1) in two cases, whereas six cases exhibited none. Two cases had lymph node metastasis (N1), whereas six cases had no lymph node metastasis. Histological grading was G1 in all cases.

**TABLE 2:** Pathological characteristics of NETs in resected stomach (surgical specimen).

	*n* = 8
Size of the largest tumor, median (range), mm	5 (1.5–8)
Number of tumors, *n* (%)	
<10	4 (50.0)
10–20	2 (25.0)
>20	2 (25.0)
Depth of the deepest tumor invasion, *n* (%)	
pT1a (M)	2 (25.0)
pT1b (SM)	6 (50.0)
Lymphovascular invasion, *n* (%)	
Ly 1, V1	0 (0.0)
Ly 0, V1	2 (25.0)
Ly 1, V0	0 (0.0)
Ly 0, V0	6 (75.0)
Lymph node metastasis, *n* (%)	
N1	2 (25.0)
N0	5 (62.5)
No lymph node dissection	1 (12.5)
Histological grading, *n* (%)	
G1	8 (100)
G2	0 (0.0)

### 3.3 Postoperative Clinical Outcomes of Antrectomy

**FIGURE 3:**
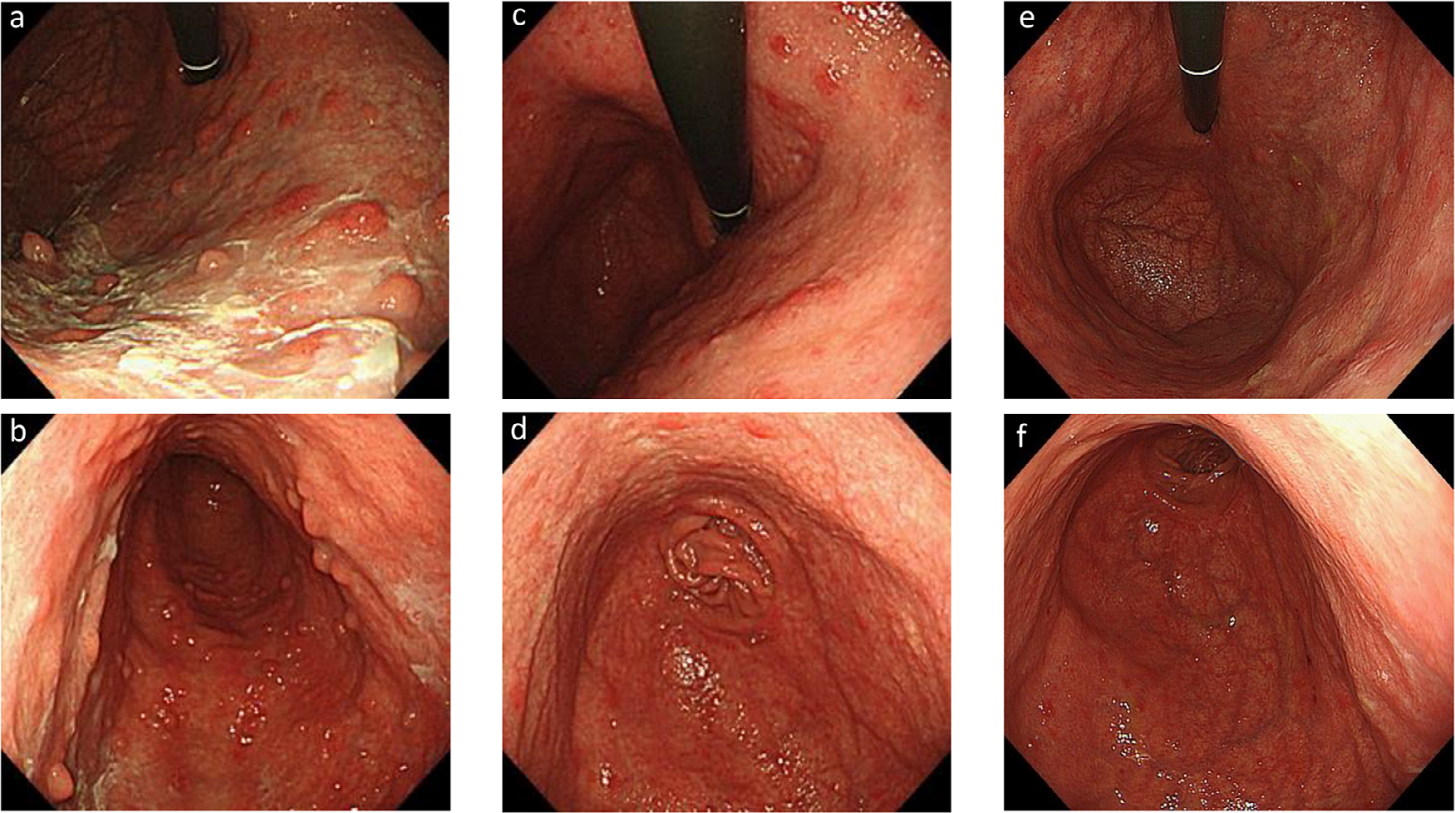
Representative case of tumor regression (case number 5). (a and b) Esophagogastroduodenoscopy in retroflex (a) and antegrade (b) views showed pre‐antrectomy findings. Multiple T1‑GNETs are observed from the gastric body to the fornix. (c and d) Esophagogastroduodenoscopy in retroflex (c) and antegrade (d) views showed 13 months after antrectomy findings. Multiple T1‑GNETs exhibited a significant regression compared to pre‐antrectomy. (e and f) Esophagogastroduodenoscopy in retroflex (c) and antegrade (d) views showed 62 months after antrectomy findings. Multiple T1‑GNETs were slightly remnant; however, they exhibited further regression and were barely noticeable.

**FIGURE 4:**
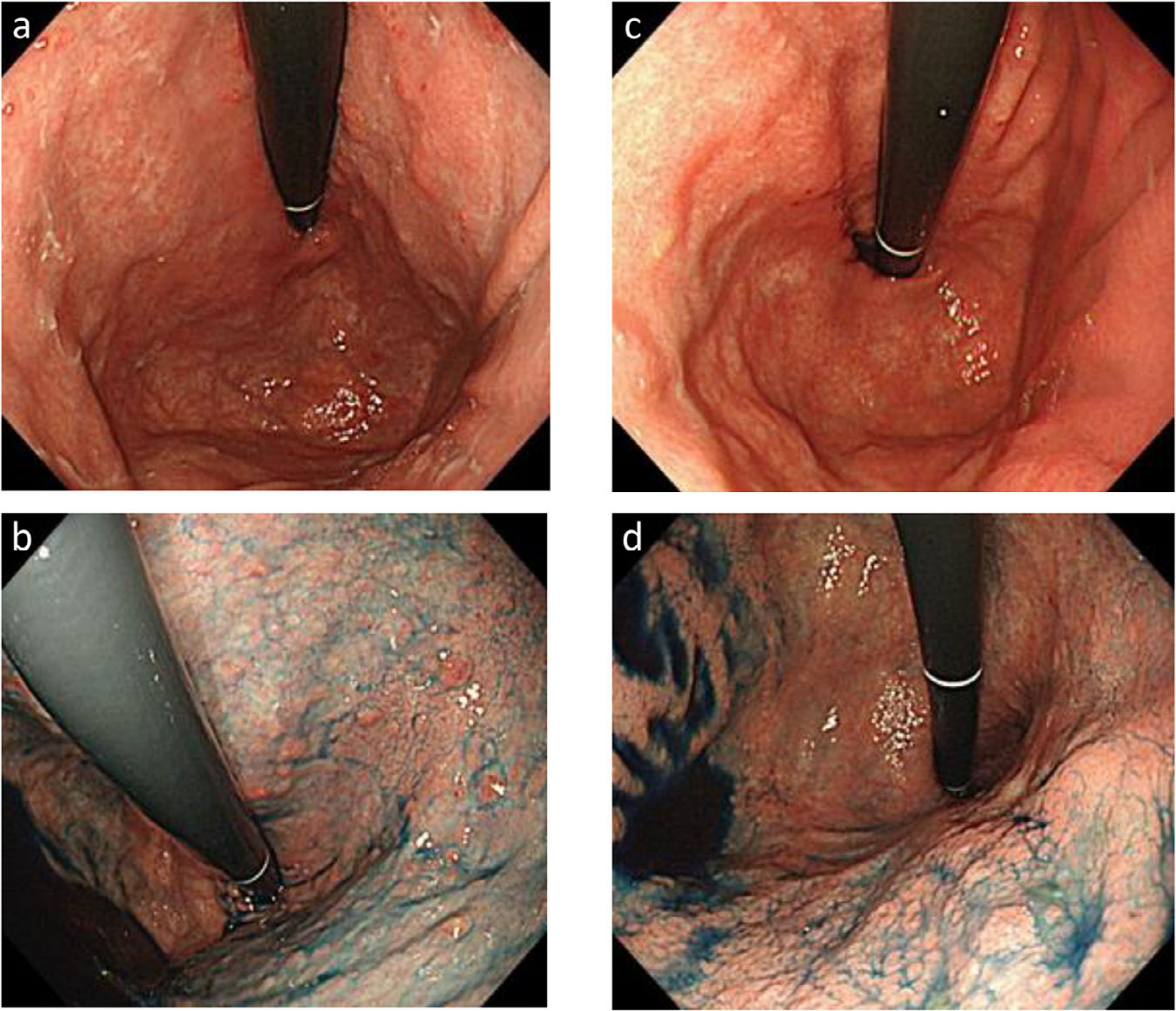
Representative case of tumor disappearance (case number 6). (a and b) Esophagogastroduodenoscopy with white light imaging (a) and indigo carmine dye spraying (b) in retroflex view showed pre‐antrectomy findings. Multiple T1‑GNETs were observed from the gastric body to the fornix. (c and d) Esophagogastroduodenoscopy with white light imaging (c) and indigo carmine dye spraying (d) in retroflex view showed 7 months after antrectomy findings. Multiple T1‑GNETs disappeared, and no residual tumor was observed.

Table 3 shows the postoperative clinical outcomes of antrectomy. The median follow‐up period was 56 months (range, 14–72 months), and the median serum gastrin levels of all patients were 68 pg/mL (range, 25–120 pg/mL). Residual tumors in a gastric remnant after antrectomy demonstrated regression in two and disappearance in six patients (Figures 3 and 4). The median period between antrectomy and tumor disappearance was 11 months (range, 5–39 months). There was no recurrence or cause‐specific death in any patient. Table 4 shows the summary of the eight cases.

**TABLE 3:** Clinical outcomes after antrectomy.

	*n* = 8
Follow‐up period after antrectomy, median (range), months	56 (14–72)
Serum gastrin levels, median (range), pg/mL	
Post‐antrectomy	68 (25–120)
Post‐antrectomy status of residual tumors, *n* (%)	
Stable	0 (0.0)
Progression	0 (0.0)
Regression	2 (25.0)
Disappearance	6 (75.0)
Period of until disappearance, median (range), months	
After antrectomy	11 (5–39)
Recurrence, *n* (%)	0 (0.0)
Tumor‐related death, *n* (%)	0 (0.0)

**TABLE 4:** Summary of the eight cases.

Case	Age (years)	Gender	Number	Size (mm)	ER	Depth	LVI	LNM	WHO	Gastrin (pg/mL)	Follow‐up (months)	Post‐antrectomy
1	44	M	>20	8	Yes	SM	N	N0	G1	5600 → 110	24	Regression
2	66	F	<10	6	Yes	M	N	N0	G1	2400 → N/A	30	Disappearance
3	54	M	>20	8	Yes	M	N	N0	G1	3300 → 78	60	Disappearance
4	52	F	>20	4	No	SM	N	N0	G1	6800 → 120	72	Disappearance
5	65	F	10–20	4	No	SM	N	N0	G1	3900 → 54	62	Regression
6	46	F	<10	6	Yes	SM	P (V1)	N1	G1	8000 → 68	60	Disappearance
7	67	F	>20	6	No	SM	N	N1	G1	3000 → 26	52	Disappearance
8	70	F	>20	6	Yes	SM	P (V1)	N0	G1	11,000 → 25	14	Disappearance

Abbreviations: depth, depth of tumor invasion; ER, pre‐antrectomy endoscopic resection; F, female; follow‐up, follow‐up period; gastrin, serum gastrin levels (pre‐antrectomy → post‐antrectomy); LNM, lymph node metastasis; LVI, lymphovascular invasion; M, male; M, mucosa; N, negative; N/A, not available; number, number of tumor (whole stomach); P, positive; post‐antrectomy, post‐antrectomy status of residual tumors; size, size of largest tumor (whole stomach); SM, submucosa; WHO, World Health Organization classification.

## 4 Discussion

In this study, we evaluated the residual T1‑GNETs in the gastric remnant after antrectomy, demonstrating regression or disappearance in all cases. In addition, the combination of antrectomy and ER has enabled reliable and minimally invasive management. These findings indicate that this approach may represent a potential organ‐preserving strategy for patients with multiple T1‑GNETs.

In our study, residual NETs in the gastric remnant regressed in 25% and disappeared in 75% of all cases, without any recurrence. At the first endoscopic follow‐up (median, 7 months; range, 4–30 months), regression or disappearance was observed. A median period for tumor disappearance was 11 months (range, 5–39 months). In previous studies, T1‑GNETs regressed or disappeared in 89%–100% of post‐antrectomy cases [10, 14, 15] (disappeared in 9/10 [10], disappeared in 8/8 [14], regression or disappeared in 17/19 [15]), with similarly favorable treatment outcomes to those of the current study. The normalization of serum gastrin levels, achieved through removing gastrin‐producing pyloric glands by antrectomy, may contribute to tumor regression or disappearance [10, 11, 12]. In this study, gastrin levels decreased drastically in all cases post‐antrectomy from 4800 to 67 pg/mL (median). These findings are consistent with previously published reports [13, 14, 15, 16, 17, 18]. Although antrectomy is mentioned as a potential treatment option in both the Japanese and the NCCN guidelines, the rarity of the disease has limited the accumulation of high‐quality evidence sufficient to establish it as a recommended standard therapy. In this context, the present study provides additional clinical data that may contribute to the evidence base for the management of T1‑GNETs.

On the basis of the treatment approach of our institution, tumors measuring 6–9 mm in diameter located in the proximal side of the estimated resection edge of antrectomy were endoscopically removed before surgery. Therefore, the residual tumors in the gastric remnant should be <5 mm after antrectomy. Previous reports have indicated that tumor size >10 mm is a risk factor for metastasis [26, 27, 28]. The Japanese guidelines recommend gastrectomy with lymph node dissection for those tumors >10 mm [7]. Although, similar to our findings, cases of lymph node metastasis arising from lesions ≤5 mm have been reported [22], it is widely accepted that tumor size is positively correlated with the risk of lymph node metastasis [22, 26, 28]. In the most recent Japanese guidelines [29], surveillance is weakly recommended for lesions ≤10 mm. Therefore, although the risk of lymph node metastasis in lesions ≤5 mm is not negligible, it is considered to be relatively low. Taken together with our finding that 75% of lesions disappeared following antrectomy, a strategy of leaving lesions ≤5 mm untreated may be considered acceptable. Moreover, given that 62.5% of patients had more than 20 lesions, complete resection of all residual lesions would not be feasible in clinical practice. In addition, tumor sizes <5 mm were reported to demonstrate minimal risk of metastasis [26, 27, 28]. Thus, we determined that tumors measuring 6–9 mm in diameter should be resected considering their increased risk of metastasis and the potential to grow to 10 mm. As previous reports have documented cases in which residual lesions did not regress or disappear following antrectomy [15, 30] and lesions exceeding 10 mm may require TG according to current guidelines, pre‐antrectomy ER for lesions approaching this threshold is considered a preferable strategy to avoid the need for such an extensive surgical procedure. Therefore, this treatment approach manages the possible risk in future resection of gastric remnant. Furthermore, antrectomy was performed, including the site of the endoscopically resected tumor, due to positive LVI necessitating completion surgery following non‐curative ER. Pre‐antrectomy ER was instrumental in determining the optimal resection margin for gastrectomy.

However, TG has frequently been performed for patients with multiple T1‑GNETs due to concerns regarding residual tumors [22]. The necessity of TG for complete resection of all T1‑GNETs remains controversial, given its disadvantages, including high invasiveness, risk of complications, and postoperative decline in QOL. Compared to DG, TG has been associated with significantly worse general condition physical functioning, and greater symptom burden [31]. Prior studies using the Postgastrectomy Syndrome Assessment Scale (PGSAS)‐45 have also demonstrated poorer outcomes in body weight, reflux symptoms, meal‐related distress, and overall satisfaction with meals and daily life [32], underscoring the clinical importance of avoiding TG. Consequently, TG may be an overtreatment considering the favorable prognosis of T1‑GNETs, the low risk of metastasis in small tumors [26, 27, 28], and the effectiveness of tumor‐shrinkage on residual tumors by antrectomy, including our results. Our institution's treatment approach, which combines antrectomy with pre‐antrectomy ER when necessary, does not require the resection of all tumors, thereby representing a less invasive alternative to TG. This is a notable feature of this treatment strategy. However, as this study lacks a comparative group, the relative benefits in the combination of antrectomy and ER compared with TG cannot be determined. Furthermore, postoperative outcomes such as body weight, nutritional status, and QOL were not evaluated, and it remains unclear whether gastric preservation leads to better clinical outcomes. These limitations should be considered when interpreting the potential clinical significance of this organ‐preserving approach. Additionally, the combination of ER and antrectomy has never been reported. ER has the advantage of minimal invasiveness, whereas comprehensive removal of all T1‑GNETs via ER is often challenging in cases with numerous lesions. In the current study, 75% (6/8) of cases exhibited more than 10 T1‐GNET lesions. Forty‐three T1‑GNETs were pathologically confirmed in the resected specimens by antrectomy in a very extensive case. Therefore, the cases with multiple T1‑GNETs in this study were considered good indications for antrectomy. Other treatments, such as somatostatin analog (SSA) therapies including octreotide, lanreotide, and everolimus, are included in the NCCN guidelines [4]; however, the number of published reports remains limited [33, 34, 35, 36, 37, 38, 39]. A meta‐analysis of three studies on SSA therapies reported a cumulative recurrence rate of 30.2% (95% CI 13.1–50.6) after 34 months [34]. In a study analyzing the macroscopic and histopathological changes in five cases of GNETs 5 years after discontinuing octreotide, progression of GNETs was observed in all cases. Thus, SSA presents a much higher risk of recurrence and increased costs due to long‐term administration compared to antrectomy.

This study has several limitations. First, this was a retrospective study conducted at a single institution with a small sample size due to the rarity of multiple T1‑GNETs and variability in treatment approaches. Therefore, collaborative research across multiple institutions will be crucial in the future. In addition, broad definition was applied to antrectomy in the current study. A larger cohort study comparing conventional antrectomy to DG with lymph node dissection is needed to evaluate the importance of lymph node dissection for T1‑GNETs. Previous reports and this study have suggested that resecting the pyloric glands to normalize gastrin levels may be the potential management of multiple T1‑GNETs. However, this approach has not been established and requires further validation.

Finally, the method used to evaluate residual lesions in the gastric remnant represents another limitation. Although biopsy was performed when residual disease was suspected, the assessment of tumor regression or disappearance relied primarily on endoscopic findings. Therefore, as biopsy was not performed for all lesions, the possibility of microscopic residual disease cannot be entirely excluded.

In conclusion, we report the clinical outcomes of patients with residual T1‑GNETs who underwent antrectomy. On the basis of our findings, antrectomy directly manages tumors and may indirectly prevent recurrence and induce regression of tumors in the gastric remnant. These findings suggest that the combination of antrectomy and ER was associated with tumor regression and may represent a potential organ‐preserving approach for patients with multiple T1‑GNETs.

## Author Contributions

Yuki Morita and Ken Namikawa contributed equally to this work as first authors. Toshiaki Hirasawa substantially contributed to the conception of the work. Yuki Morita, Ken Namikawa, and Toshiaki Hirasawa made contributions to collecting and analyzing clinical data. Yuki Morita wrote the first draft of the manuscript, whereas Ken Namikawa and Toshiaki Hirasawa designed the structure of the manuscript and made substantial contributions to the manuscript editing. All authors critically reviewed and revised the manuscript draft and approved the final version for submission.

## Funding

The authors have nothing to report.

## Ethics Statement

This study was approved by the Institutional Review Board of the Cancer Institute Hospital, Japanese Foundation for Cancer Research (2023‐GB‐013). In addition, the study was conducted in accordance with the ethical standards outlined in the 1964 Declaration of Helsinki and its subsequent amendments.

## Consent

Consent was waived.

## Conflicts of Interest

The authors declare no conflicts of interest.

## References

[1] I. D. Nagtegaal , R. D. Odze , D. S. Klimstra , et al., “The 2019 WHO Classification of Tumours of the Digestive System,” Histopathology 76, no. 2 (2020): 182–188, 10.1111/his.13975. 31433515 PMC7003895

[2] G. Rindi , O. Luinetti , M. Cornaggia , C. Capella , and E. Solcia , “Three Subtypes of Gastric Argyrophil Carcinoid and the Gastric Neuroendocrine Carcinoma: A Clinicopathologic Study,” Gastroenterology 104, no. 4 (1993): 994–1006, 10.1016/0016-5085(93)90266-F. 7681798

[3] G. Rindi and F. Inzani , “Neuroendocrine Neoplasm Update: Toward Universal Nomenclature,” Endocrine‐Related Cancer 27, no. 6 (2020): R211–R218, 10.1530/ERC-20-0036. 32276263

[4] M. H. Shah , W. S. Goldner , A. B. Benson , et al., “Neuroendocrine and Adrenal Tumors, Version 2.2021, NCCN Clinical Practice Guidelines in Oncology,” Journal of the National Comprehensive Cancer Network 19, no. 7 (2021): 839–868, 10.6004/jnccn.2021.0032. 34340212

[5] R. A. Gladdy , V. E. Strong , D. Coit , et al., “Defining Surgical Indications for Type I Gastric Carcinoid Tumor,” Annals of Surgical Oncology 16, no. 11 (2009): 3154–3160, 10.1245/s10434-009-0687-y. 19727959

[6] G. Delle Fave , D. O'Toole , A. Sundin , et al., “ENETS Consensus Guidelines Update for Gastroduodenal Neuroendocrine Neoplasms,” Neuroendocrinology 103, no. 2 (2016): 119–124, 10.1159/000443168. 26784901

[7] T. Ito , T. Masui , and I. Komoto , Clinical Practice Guidelines for Gastroenteropancreatic Neuroendocrine Neoplasms (NEN) 2019, 2nd ed. (Japanese Neuroendocrine Tumor Society, 2019).

[8] M. Itsuno , H. Watanabe , M. Iwafuchi , et al., “Multiple Carcinoids and Endocrine Cell Micronests in Type A Gastritis. Their Morphology, Histogenesis, and Natural History,” Cancer 63, no 5 (1989): 881–890. 2644016 10.1002/1097-0142(19890301)63:5<881::aid-cncr2820630515>3.0.co;2-k

[9] M. H. Kulke , M. H. Shah , A. B. Benson , et al., “Neuroendocrine Tumors, Version 1.2015,” Journal of the National Comprehensive Cancer Network 13, no. 1 (2015): 78–108, 10.6004/jnccn.2015.0011. 25583772

[10] K. Borch , B. Ahrén , H. Ahlman , S. Falkmer , G. Granérus , and L. Grimelius , “Gastric Carcinoids: Biologic Behavior and Prognosis After Differentiated Treatment in Relation to Type,” Annals of Surgery 242, no. 1 (2005): 64–73, 10.1097/01.sla.0000167862.52309.7d. 15973103 PMC1357706

[11] G. F. Dakin , R. R. Warner , A. Pomp , B. Salky , and W. B. Inabnet , “Presentation, Treatment, and Outcome of Type 1 Gastric Carcinoid Tumors,” Journal of Surgical Oncology 93, no. 5 (2006): 368–372, 10.1002/jso.20468. 16550587

[12] D. Thomas , A. V. Tsolakis , S. Grozinsky‐Glasberg , et al., “Long‐Term Follow‐Up of a Large Series of Patients With Type 1 Gastric Carcinoid Tumors: Data From a Multicenter Study,” European Journal of Endocrinology 168, no. 2 (2013): 185–193, 10.1530/EJE-12-0836. 23132699

[13] M. Hoshino , N. Omura , F. Yano , et al., “Usefulness of Laparoscope‐Assisted Antrectomy for Gastric Carcinoids With Hypergastrinemia,” Hepato‐Gastroenterology 57, no. 98 (2010): 379–382. 20583448

[14] J. Ozao‐Choy , K. Buch , J. A. Strauchen , R. R. P. Warner , and C. M. Divino , “Laparoscopic Antrectomy for the Treatment of Type I Gastric Carcinoid Tumors,” Journal of Surgical Research 162, no. 1 (2010): 22–25, 10.1016/j.jss.2010.01.005. 20421108

[15] H. E. Jenny , P. A. Ogando , K. Fujitani , R. R. Warner , and C. M. Divino , “Laparoscopic Antrectomy: A Safe and Definitive Treatment in Managing Type 1 Gastric Carcinoids,” American Journal of Surgery 211, no. 4 (2016): 778–782, 10.1016/j.amjsurg.2015.08.040. 26992358

[16] B. I. Hirschowitz , J. Griffith , D. Pellegrin , and O. W. Cummings , “Rapid Regression of Enterochromaffin‐Like Cell Gastric Carcinoids in Pernicious Anemia After Antrectomy,” Gastroenterology 102, no. 4 (1992): 1409–1418. 1551550

[17] F. E. Eckhauser , R. V. Lloyd , N. W. Thompson , S. E. Raper , and A. I. Vinik , “Antrectomy for Multicentric, Argyrophil Gastric Carcinoids: A Preliminary Report,” Surgery 104, no. 6 (1988): 1046–1053. 3194832

[18] J. Kitadani , T. Ojima , K. Hayata , et al., “Single‐Incision Laparoscopic Antrectomy for Type I Gastric Neuroendocrine Tumor: A Case Report,” Surgical Case Reports 7 (2021): 15, 10.1186/s40792-021-01109-7. 33433761 PMC7803843

[19] D. Ravizza , M. Giunta , I. Sala , et al., “Gastric Neuroendocrine Tumors: 20‐Year Experience in a Reference Center,” Journal of Neuroendocrinology 36, no. 12 (2024): e13440, 10.1111/jne.13440. 39191460

[20] J. H. Noh , D. H. Kim , H. Yoon , et al., “Clinical Outcomes of Endoscopic Treatment for Type 1 Gastric Neuroendocrine Tumor,” Journal of Gastrointestinal Surgery 25, no. 10 (2021): 2495–2502, 10.1007/s11605-021-04997-0. 33825119

[21] Y. Sato , H. Imamura , Y. Kaizaki , et al., “Management and Clinical Outcomes of Type I Gastric Carcinoid Patients: Retrospective, Multicenter Study in Japan,” Digestive Endoscopy 26, no. 3 (2014): 377–384, 10.1111/den.12197. 24188531

[22] K. Namikawa , T. Kamada , J. Fujisaki , et al., “Clinical Characteristics and Long‐Term Prognosis of Type 1 Gastric Neuroendocrine Tumors in a Large Japanese National Cohort,” Digestive Endoscopy 35, no. 6 (2023): 757–766, 10.1111/den.14529. 36721901 PMC12136246

[23] L. Lundell , “Acid Secretion and Gastric Surgery,” Digestive Diseases 29, no. 5 (2011): 487–490, 10.1159/000331516. 22095015

[24] T. Kamada , H. Watanabe , T. Furuta , et al., “Diagnostic Criteria and Endoscopic and Histological Findings of Autoimmune Gastritis in Japan,” Journal of Gastroenterology 58, no. 3 (2023): 185–195, 10.1007/s00535-022-01954-9. 36855000 PMC9998601

[25] Japanese Gastric Cancer Association , 2021, Japanese Gastric Cancer Treatment Guidelines 2018 (5th Edition), Gastric Cancer 24, no. 1: 1–21, 10.1007/s10120-020-01042-y. 32060757 PMC7790804

[26] A. Vanoli , S. La Rosa , E. Miceli , et al., “Prognostic Evaluations Tailored to Specific Gastric Neuroendocrine Neoplasms: Analysis of 200 Cases With Extended Follow‐Up,” Neuroendocrinology 107, no. 2 (2018): 114–126, 10.1159/000489902. 29895024

[27] S. Grozinsky‐Glasberg , D. Thomas , J. R. Strosberg , et al., “Metastatic Type 1 Gastric Carcinoid: A Real Threat or Just a Myth?” World Journal of Gastroenterology 19, no. 46 (2013): 8687–8695, 10.3748/wjg.v19.i46.8687. 24379587 PMC3870515

[28] C.‐S. Chung , C.‐L. Tsai , Y.‐Y. Chu , et al., “Clinical Features and Outcomes of Gastric Neuroendocrine Tumors After Endoscopic Diagnosis and Treatment: A Digestive Endoscopy Society of Taiwan (DEST),” Medicine (Baltimore) 97, no. 38 (2018): e12101, 10.1097/MD.0000000000012101. 30235663 PMC6160255

[29] Japanese Neuroendocrine Tumor Society (JNETS) , Clinical Practice Guidelines for Gastroenteropancreatic Neuroendocrine Neoplasms (NEN) 2026, 3rd ed. (Kanehara Shuppan, 2026).

[30] S. V. Murugesan , I. A. Steele , R. Dimaline , et al., “Correlation Between a Short‐Term Intravenous Octreotide Suppression Test and Response to Antrectomy in Patients With Type‐1 Gastric Neuroendocrine Tumours,” European Journal of Gastroenterology & Hepatology 25, no. 4 (2013): 474–481, 10.1097/MEG.0b013e32835cec52. 23249603

[31] J. Yu , Z. Wang , H. Yang , et al., “Long‐Term Health‐Related Quality of Life in Patients With Gastric Cancer After Total or Distal Gastrectomy: A Propensity Score‐Matched Cohort Study,” International Journal of Surgery 109, no. 11 (2023): 3283–3293, 10.1097/JS9.0000000000000620. 37526103 PMC10651271

[32] M. Takahashi , M. Terashima , H. Kawahira , et al., “Quality of Life After Total vs Distal Gastrectomy With Roux‐en‐Y Reconstruction: Use of the Postgastrectomy Syndrome Assessment Scale‐45,” World Journal of Gastroenterology 23, no. 11 (2017): 2068–2076, 10.3748/wjg.v23.i11.2068. 28373774 PMC5360649

[33] S. Grozinsky‐Glasberg , G. Kaltsas , C. Gur , et al., “Long‐Acting Somatostatin Analogues are an Effective Treatment for Type 1 Gastric Carcinoid Tumours,” European Journal of Endocrinology 159, no. 4 (2008): 475–482, 10.1530/EJE-08-0420. 18662970

[34] R. E. Rossi , P. Invernizzi , V. Mazzaferro , and S. Massironi , “Response and Relapse Rates After Treatment With Long‐Acting Somatostatin Analogs in Multifocal or Recurrent Type‐1 Gastric Carcinoids: A Systematic Review and Meta‐Analysis,” United European Gastroenterology Journal 8, no. 2 (2020): 140–147, 10.1177/2050640619890465. 32213066 PMC7079271

[35] D. Campana , D. Ravizza , P. Ferolla , et al., “Clinical Management of Patients With Gastric Neuroendocrine Neoplasms Associated With Chronic Atrophic Gastritis: A Retrospective, Multicentre Study,” Endocrine 51, no. 1 (2016): 131–139, 10.1007/s12020-015-0584-z. 25814125

[36] D. Campana , F. Nori , R. Pezzilli , et al., “Gastric Endocrine Tumors Type I: Treatment With Long‐Acting Somatostatin Analogs,” Endocrine‐Related Cancer 15, no. 1 (2008): 337–342, 10.1677/ERC-07-0251. 18310299

[37] V. Fykse , A. K. Sandvik , G. Qvigstad , S. E. Falkmer , U. Syversen , and H. L. Waldum , “Treatment of ECL Cell Carcinoids With Octreotide LAR,” Scandinavian Journal of Gastroenterology 39, no. 7 (2004): 621–628, 10.1080/00365520410005225. 15370681

[38] C. S. Jianu , R. Fossmark , U. Syversen , Ø. Hauso , V. Fykse , and H. L. Waldum , “Five‐Year Follow‐Up of Patients Treated for 1 Year With Octreotide Long‐Acting Release for Enterochromaffin‐Like Cell Carcinoids,” Scandinavian Journal of Gastroenterology 46, no. 4 (2011): 456–463, 10.3109/00365521.2010.539255. 21133821

[39] M. S. Khuroo , M. S. Khuroo , and N. S. Khuroo , “Treatment of Type I Gastric Neuroendocrine Tumors With Somatostatin Analogs,” Journal of Gastroenterology and Hepatology 25, no. 3 (2010): 548–554, 10.1111/j.1440-1746.2009.06131.x. 20074162

